# Conservative Therapy for Nonspecific Granuloma of the Larynx Using a Beclomethasone Dipropionate Inhaler

**DOI:** 10.1155/DTE.3.93

**Published:** 1996

**Authors:** Masahiro Kawaida, Hiroyuki Fukuda, Yoshihisa Kawasaki, Akihiro Shiotani, Naoyuki Kohno

**Affiliations:** 1 Department of Otolaryngology Tokyo Metropolitan Ohtsuka Hospital Tokyo, Japan 2-8-1 Minamiohtsuka, Toshima-ku Tokyo 170 Japan; 2 Department of Otolaryngology Keio University School of Medicine Tokyo, Japan 35 Shinanomachi, Shinjuku-ku Tokyo 160 Japan; 3 Department of Otolaryngology Juntendo University School of Medicine Tokyo, Japan 2-1-1 Hongo, Bunkyo-ku Tokyo 113 Japan

## Abstract

Nonspecific granuloma of the larynx is a benign tumor that usually occurs on the posterior glottis. Conventional treatment for this laryngeal lesion has consisted of surgical resection. However, this lesion has a strong tendency to recur postoperatively and may require multiple repeated operative procedures. Conservative treatment, consisting mainly of beclomethasone dipropionate inhalation therapy, was instituted in 20 cases with good results. We discussed the effect of beclomethasone dipropionate on this lesion as well as the etiology of this disease.


*Diagnostic and Therapeutic Endoscopy*, 1996, Vol. 3, pp. 93–98
Reprints available directly from the publisher
Photocopying permitted by license only

© 1996 OPA (Overseas Publishers Association)
Amsterdam B.V. Published in The Netherlands
by Harwood Academic Publishers
Printed in Malaysia

### Conservative Therapy for Nonspecific Granuloma of the Larynx Using a Beclomethasone Dipropionate Inhaler

MASAHIRO KAWAIDA[a,*], HIROYUKI FUKUDA[b], YOSHIHISA KAWASAKI [b], AKIHIRO SHIOTANI [b] and NAOYUKI KOHNO [c]

[a]*Department of Otolaryngology, Tokyo Metropolitan Ohtsuka Hospital, Tokyo, Japan 2-8-1, Minamiohtsuka, Toshima-ku, Tokyo 170, Japan,*
[b]*Department of Otolaryngology, Keio University School of Medicine, Tokyo, Japan 35 Shinanomachi, Shinjuku-ku, Tokyo 160, Japan,*
[c]*Department of Otolaryngology, Juntendo University School of Medicine, Tokyo, Japan 2-1-1, Hongo, Bunkyo-ku, Tokyo 113, Japan*

*(Received 21 March 1996; In final form 26 June 1996)*

**Nonspecific granuloma of the larynx is a benign tumor that usually occurs on the posterior glottis. Conventional treatment for this laryngeal lesion has consisted of surgical resection. However, this lesion has a strong tendency to recur postoperatively and may require multiple repeated operative procedures. Conservative treatment, consisting mainly of beclomethasone dipropionate inhalation therapy, was instituted in 20 cases with good results. We discussed the effect of beclomethasone dipropionate on this lesion as well as the etiology of this disease.**

*Keywords:* Nonspecific granuloma, larynx, conservative therapy, beclomethasone dipropionate, inhalation therapy

### INTRODUCTION

Nonspecific granuloma of the larynx is a benign tumor that usually occurs on the posterior glottis. Recurrences frequently occur even when these granulomas are surgically resected and it is not rare for multiple repeated operations to be required. Therefore, conservative treatment using primarily a beclomethasone dipropionate inhaler (Aldecine® inhaler) was instituted in 20 cases of nonspecific granuloma of the larynx with good results. The results are discussed in this paper, and we have attempted to explain the mechanism of therapeutic efficacy and the etiology of the disease.

### SUBJECTS

Among the patients with nonspecific granuloma of the larynx who presented to the ENT clinic of Tokyo Metropolitan Ohtsuka Hospital in the 7 years between April 1988 and March 1995, the 20 patients who underwent conservative therapy and were followed up for 6 months were selected as subjects of this study. Clinical characteristics and therapeutic results of these patients were evaluated (Table I). The subjects included 3 patients with post-intubation granuloma that developed after endotracheal intubation and 17 patients with idiopathic granuloma unrelated to endotracheal intubation. Median patient age at time of the

*Corresponding author. Tel.: +81-3-3941-3211. Fax: +81-3-3941-9557.

TABLE I Clinical characteristics and therapeutic results of beclomethasone dipropionate inhalation therapy

| Patient No. | Age at Diagnosis (yr) | Sex | Chief Complaints | Idiopathic or Post-intubation | Site | Time of Disappearance (months) | Past History | Efficacy |
|---|---|---|---|---|---|---|---|---|
| 1 | 66 | Male | Cough hoarseness | Idiopathic | Left | 3 months | Diabetes mellitus | E |
| 2 | 71 | Male | AST, cough hoarseness | Idiopathic | Left | 1 month | (–) | E |
| 3 | 64 | Female | Cough hoarseness | Post-intubation | Left | 3 months and 2 weeks | Colon carcinoma | G |
| 4 | 42 | Male | AST, cough | Idiopathic | Right | 1 month and 2 weeks | (–) | E |
| 5 | 21 | Female | AST, hoarseness | Post-intubation | Right | 5 months | Head injury | G |
| 6 | 44 | Male | AST, cough hoarseness | Idiopathic | Left | 8 months | Granuloma reaction | F |
| 7 | 50 | Male | Cough hoarseness | Idiopathic | Left | 3 months | (–) | E |
| 8 | 55 | Female | AST, cough | Idiopathic | Left | 3 months | (–) | E |
| 9 | 44 | Male | AST | Idiopathic | Left | 24 months | Granuloma resection | F |
| 10 | 39 | Male | AST, hoarseness | Idiopathic | Left | 5 months | (–) | G |
| 11 | 52 | Male | AST, | Idiopathic | Left | 5 months | (–) | G |
| 12 | 45 | Male | AST, cough | Idiopathic | Right | 4 months | (–) | G |
| 13 | 49 | Male | AST, | Idiopathic | Right | 2 months | (–) | E |
| 14 | 33 | Male | AST, | Idiopathic | Left | Residual on 21 months | Granuloma resection | P |
| 15 | 50 | Male | AST, cough hoarseness | Post-intubation | Bilateral | 3 months | Vertebral tumor | E |
| 16 | 46 | Male | AST, cough hoarseness | Idiopathic | Right | Residual on 12 months | (–) | P |
| 17 | 49 | Male | AST, | Idiopathic | Left | Residual on 12 months | (–) | P |
| 18 | 52 | Male | AST, cough | Idiopathic | Right | Residual on 7 months | (–) | P |
| 19 | 74 | Male | AST, cough hoarseness | Idiopathic | Right | 2 months | (–) | E |
| 20 | 50 | Male | Hoarsen | Idiopathic | Left | 2 months | (–) | E |

AST: abnormal sensation in the throat, E: excellent, G: good, F: fair, P: poor initial examination was 49.8 years, with a range from 21 to 74 years. There were 17 men and 3 women. All of the granulomas were located on the vocal process of the arytenoid cartilage. The granulomas had developed on the right side in 7 cases, left side in 12 cases and bilaterally in 1 case. Common symptoms included an abnormal sensation in the throat, cough and hoarseness. In some cases, coughing and throat-clearing appeared first, followed by an abnormal sensation in the throat and hoarseness. Three patients, all of them with idiopathic granuloma, were referred to our clinic because of recurrence following resection at another hospital, and the definitive histopathological diagnosis was nonspecific granuloma of the larynx.

### TREATMENT METHOD AND ASSESSMENT OF EFFICACY

All of the subjects underwent beclomethasone dipropionate inhalation therapy as outpatients. The inhaler contained a suspension of beclomethasone dipropionate, whose structural formula is shown in Figure. 1, sealed in an atomizer. The atomizer releases approximately 0.05 mg per spray. Two inhalations (a dose of approximately 0.1 mg) were given four times a day based on the protocol for the treatment of bronchial asthma. All of the patients were instructed to refrain from unnecessary coughing and loud talking.

Therapeutic efficacy was assessed by local findings in the larynx observed with a laryngoendoscope. We established our own assessment criteria for therapeutic efficacy, classifying the results as excellent, good, fair, or poor (Table II).

### RESULTS

Table I shows the results of the assessment of the therapeutic efficacy. The response was excellent in 9 patients, good in 5, fair in 2, and poor in 4. Fourteen patients had an excellent or good response, for an efficacy rate of 70%. Six patients had a fair or poor response, but three of them had a recurrence following surgical resection at other hospitals prior to referral to our clinic. All of the lesions eventually resolved except 4 patients who had showed a poor response, and none of the 16 patients with completely resolved lesions have experienced subsequent recurrences.

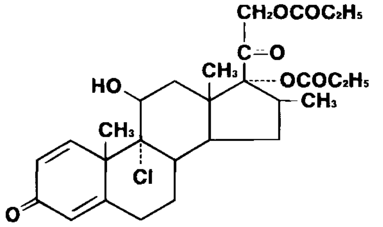

FIGURE 1 Structural formula of beclomethasone of dipropionate.

TABLE II Criteria for assessment of efficacy of beclomethasone dipropionate inhalation therapy in nonspecific granuloma of the larynx

| |
|---|
| Excellent: Complete disappearance of the lesion within 3 months of the start of inhalation therapy |
| Good: Reduction in size of the lesion within 3 months of the start to inhalation therapy and complete disappearance of the lesion within 6 months |
| Fair: Reduction in size of the lesion within 3 months, but residual lesion observed after 6 months of inhalation therapy |
| Poor: No change after 3 months of inhalation therapy |

The therapeutic efficacy was established by assessment based on laryngoendoscopic findings.

### CASE PRESENTATION

One of the 20 patients who had an excellent response is presented here. Patient number 7 was a 50-year-old man whose chief complaint was coughing and hoarseness. He developed a mild cough in January 1990, and complained of hoarseness that made it difficult to produce a high-pitched voice in July 1990. He first presented to our clinic in August 1991. Flexible laryngofiberscopy revealed a grayish-white, spherical mass observed on the left vocal process of the arytenoid cartilage (Figure 2-a). Since the lesion appeared to be an idiopathic nonspecific granuloma of the larynx, beclomethasone dipropionate inhalation therapy was started. After approximately 1 week, his symptoms gradually improved, and after 4 weeks of inhalation therapy, the site of lesion had been reduced by approximately 50% (Figure 2-b). A reduction in size of approximately 75% was observed after 8 weeks (Figure 2-c). At the end of 12 weeks, the symptoms of coughing and hoarseness had resolved, and flexible laryngofiberscopy revealed no evidence of the lesion (Figure 2-d). The patient has not experienced any recurrences.

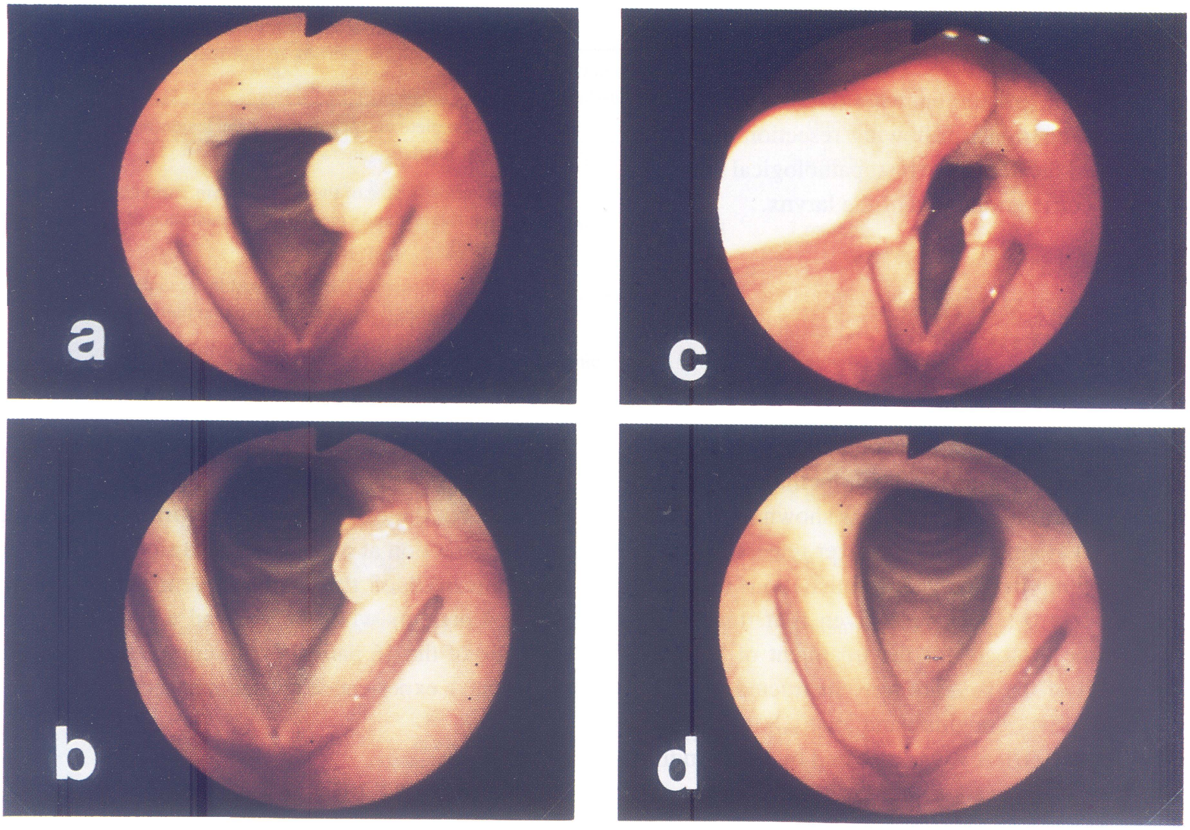

FIGURE 2 Flexible laryngofiberscopic findings in a patient with idiopathic nonspecific granuloma of the larynx. a (Initial findings), A grayish white, spherical mass observed on the left vocal process. b (After 4 weeks of treatment), The lesion has reduced in size approximately 50%. c (After 8 weeks of treatmenr), The lesion had reduced in size approximately 75%. d (After 12 weeks of treatment), The lesion had completely disappeared.

### DISCUSSION

The most common types of nonspecific granuloma of the larynx are post-intubation granuloma and idiopathic granuloma unrelated to endotracheal intubation. Surgical resection has been the treatment of first choice. Patients seldom experience recurrences after surgical resection of post-intubation granulomas. Patients with idiopathic granulomas, however, often present with repeated recurrences postoperatively [1,2]. It has been suggested that surgical resection may actually promote the development of these granulomas. Therefore, treatment of such granulomas by conservative therapy might reduce the recurrence rate.

Excessive hard attacks of the posterior glottis due to coughing and habitual throat-clearing has been reported to be a possible cause of idiopathic granuloma of the larynx [3,4]. Cough was a chief complaint in 10 of the 17 patients with idiopathic granuloma in our study. In patients without coughing or habitual throat-clearing, the most common chief complaint was an abnormal sensation in the throat, and these patients may have often cleared their throats unconsciously. Two of the three patients with post-intubation granuloma complained of coughing, and it appears that the cause may not have been only the intubation procedure itself but also excessive hard attacks of the posterior glottis during coughing as a result of airway irritation from the procedure. Thus, granulation tissue in idiopathic granuloma likely develops secondary to microtrauma and perichondritis of the vocal process of the arytenoid cartilage because of excessive hard glottal attacks caused by coughing, habitual throat-clearing, voice abuse, or esophago-pharyngeal acid reflux [5].

Once an idiopathic or post-intubation granuloma has developed, the granulomatous lesion itself may irritate the airways mechanically, inducing more coughing and throat-clearing. Natural healing, which would occur in the absence of mechanical irritation, also seems to be hindered by excessive hard attacks of the posterior glottis because of coughing and habitual throat-clearing. The traditional treatment of choice mainly has been maintenance of silence or voice therapy [3,6–8]. However, because prolonged silence is impractical for many patients, and because neoplasm cannot be excluded without a definitive histopathological diagnosis, surgical resection has become the treatment of choice.

The utility of beclomethasone dipropionate inhalation therapy in the treatment of nonspecific granuloma of the larynx was first reported in 1989 [9]. A total of 20 patients have been treated with beclomethasone dipropionate inhalation therapy in our clinic, and they comprise the subjects of the present study. The fact that the granulomatous lesion eventually disappeared in 16 patients is consistent with the efficacy of this inhalation therapy. The mechanism of action of this inhalation therapy will be discussed below.

Nonspecific granuloma of the larynx results mainly from inflammatory granulation tissue proliferation [10]. Drugs with potent anti-inflammatory action, such as corticosteroids, should be capable of inhibiting granulation and should cause the lesion to resolve. In addition, such drugs also act on the airways to suppress nonspecific inflammation and reduce airway hypersensitivity [11,12]. This inhalation therapy reduces the need for coughing and throat-clearing, thus eliminating mechanical irritation associated with excessive hard attacks of the posterior glottis. It appears that excessive hard attacks of the posterior glottis persisted in "poor response" patients, who failed to beclomethasone dipropionate inhalation therapy, such as in patients in whom habitual throat-clearing did not improve.

Beclomethasone dipropionate is applied topically, and the risk of adverse reactions occurring with corticosteroid inhalation therapy must be considered. However, few adverse reactions typically caused by systemic glucocorticoid action occur at the therapeutic dose, and the inhaler is easy for outpatient to use because of the quantitative atomizer [13–15]. All 20 patients used the inhaler on an outpatient basis with no adverse reactions.

Nonspecific granuloma of the larynx most often arises on the posterior glottis, and laryngoendoscopic diagnosis is generally easy in many cases. However, these lesions must be differentiated from neoplasms such as laryngeal carcinoma and papilloma. Therefore, when no abnormal findings exist on general examination and clinical suspicion of nonspecific granuloma is high, the treatment plan described below is used in our clinic.

Conservative therapy predominantly using a beclomethasone dipropionate inhaler is prescribed for 3 months for both idiopathic and post-intubation granuloma. In addition, unnecessary throat-clearing is discouraged, and patients are instructed to refrain from loud talking and to remain silent whenever possible. If the size of the lesion diminishes after 3 months, conservative therapy is continued for another 3 months and the patient's course is followed. If, however, the lesion has not resolved after 6 months, or if no changes occur after the first three months, the lesion is resected surgically and examined histopathologically.

To definitively evaluate the utility of beclomethasone dipropionate inhalation therapy in the treatment of nonspecific granuloma of the larynx, it seems necessary to compare its therapeutic efficacy in a group treated conservatively with inhalation therapy and a group treated conservatively without inhalation therapy. We are thus planning to increase the number of cases and perform a controlled study in two such groups. Although long-term follow-up results are also unavailable for evaluating the utility of this inhalation therapy, it appears preliminarily to be worth while prior to attempting surgical resection.

### References

[1] Benjamin, B. and Croxson, G. (1985). Vocal cord granuloma. *Ann. Otol. Rhinol. Laryngol.*, **94**, 538–541.

[2] Shin, T., Watanabe, H., Oda, M., *et al.* (1994). Contact granulomas of the Larynx. *Eur. Arch. Otorhinolaryngol.*, 251, 67–71.

[3] von Leden, H. and Moore, P. (1960). Contact ulcer of the larynx; Experimental observations. *Arch. Otolaryngol.*, **72**, 746–752.
[4] Ward, P. H., Zwitman, D., Hanson, D. *et al.* (1980). Contact ulcers and granulomas of the larynx; New insights into their etiology as a basis for more rational treatment. *Otolaryngol. Head Neck Surg.*, **88**, 262–269.
[5] McFerran, D. J., Abdullah, V., Gallimore, A. P. *et al.* (1994). Vocal process granulomata. *J. Laryngol. Otol.*, **108**, 216–220.
[6] Jackson, C. and Jackson, C. L. (1938). Contact ulcer of the larynx. *Arch. Otolaryngol.*, **22**, 1–15.
[7] Peacher, G. M. (1961). Vocal therapy for contact ulcer of the larynx; A follow-up of 70 patients. *Laryngoscope*, **71**, 37–47.
[8] Bloch, C. S., Gould, W. J. and Hirano, M. (1981). Effect of voice therapy on contact granuloma of the vocal fold. *Ann. Otol. Rhinol. Laryngol.*, **90**, 48–52.
[9] Kawasaki, Y., Fukuda, H., Kawaida, M. *et al.* (1989). Beclomethasone dipropionate inhalation therapy for granulomas of the larynx. *Otologia Fukuoka*, **35**, 604–608.
[10] Wenig, B. M. and Heffner, D. K. (1990). Contact ulcers of the larynx; A reacquaintance with the pathology on an often underdiagnosed entity. *Arch. Pathol. Lab. Med.*, **114**, 825–828.
[11] Ryan, G., Latimer, K. M., Juniper, E. F. *et al.* (1985). Effect of beclomethasone dipropionate on bronchial responsiveness to histamine in controlled nonsteroid-dependent asthma. *J. Allergy Clin. Immunol.*, **75**, 25–30.
[12] Szefler, S. J. (1991). Glucocorticoid therapy for asthma; Clinical pharmacology. *J. Allergy Clin. Immunol.*, **88**, 147–165.
[13] Brown, H. M., Storey, G. and George, W. H. S. (1972). Beclomethasone dipropionate; A new steroid aerosol for the treatment of allergic asthma. *Brit. Med. J.*, **1**, 585–590.
[14] Williams, N. H. (1981). Beclomethasone dipropionate. *Ann. Internal Med.*, **95**, 464–467.
[15] Smith, M. J. and Hadson, M. E. (1983). Effects of long term inhaled high dose beclomethasone dipropionate on adrenal function. *Thorax*, **38**, 676–681.

